# Angiotensin-converting enzyme inhibitor treatment early after myocardial infarction attenuates acute cardiac and neuroinflammation without effect on chronic neuroinflammation

**DOI:** 10.1007/s00259-020-04736-8

**Published:** 2020-03-03

**Authors:** Tobias Borchert, Annika Hess, Mario Lukačević, Tobias L. Ross, Frank M. Bengel, James T. Thackeray

**Affiliations:** Department of Nuclear Medicine, Hannover Medical School, Carl Neuberg-Str. 1, D-30625 Hannover, Germany

**Keywords:** Inflammation, Myocardial infarction, Neuroinflammation, Positron emission tomography, TSPO

## Abstract

**Purpose:**

Myocardial infarction (MI) triggers a local inflammatory response which orchestrates cardiac repair and contributes to concurrent neuroinflammation. Angiotensin-converting enzyme (ACE) inhibitor therapy not only attenuates cardiac remodeling by interfering with the neurohumoral system, but also influences acute leukocyte mobilization from hematopoietic reservoirs. Here, we seek to dissect the anti-inflammatory and anti-remodeling contributions of ACE inhibitors to the benefit of heart and brain outcomes after MI.

**Methods:**

C57BL/6 mice underwent permanent coronary artery ligation (*n* = 41) or sham surgery (*n* = 9). Subgroups received ACE inhibitor enalapril (20 mg/kg, oral) either early (anti-inflammatory strategy; 10 days treatment beginning 3 days prior to surgery; *n* = 9) or delayed (anti-remodeling; continuous from 7 days post-MI; *n* = 16), or no therapy (*n* = 16). Cardiac and neuroinflammation were serially investigated using whole-body macrophage- and microglia-targeted translocator protein (TSPO) PET at 3 days, 7 days, and 8 weeks. In vivo PET signal was validated by autoradiography and histopathology.

**Results:**

Myocardial infarction evoked higher TSPO signal in the infarct region at 3 days and 7 days compared with sham (*p* < 0.001), with concurrent elevation in brain TSPO signal (+ 18%, *p* = 0.005). At 8 weeks after MI, remote myocardium TSPO signal was increased, consistent with mitochondrial stress, and corresponding to recurrent neuroinflammation. Early enalapril treatment lowered the acute TSPO signal in the heart and brain by 55% (*p* < 0.001) and 14% (*p* = 0.045), respectively. The acute infarct signal predicted late functional outcome (*r* = 0.418, *p* = 0.038). Delayed enalapril treatment reduced chronic myocardial TSPO signal, consistent with alleviated mitochondrial stress. Early enalapril therapy tended to lower TSPO signal in the failing myocardium at 8 weeks after MI (*p* = 0.090) without an effect on chronic neuroinflammation.

**Conclusions:**

Whole-body TSPO PET identifies myocardial macrophage infiltration and neuroinflammation after MI, and altered cardiomyocyte mitochondrial density in chronic heart failure. Improved chronic cardiac outcome by enalapril treatment derives partially from acute anti-inflammatory activity with complementary benefits in later stages. Whereas early ACE inhibitor therapy lowers acute neuroinflammation, chronic alleviation is not achieved by early or delayed ACE inhibitor therapy, suggesting a more complex mechanism underlying recurrent neuroinflammation in ischemic heart failure.

**Electronic supplementary material:**

The online version of this article (10.1007/s00259-020-04736-8) contains supplementary material, which is available to authorized users.

## Introduction

Myocardial infarction (MI) remains a major contributor to morbidity, mortality, and healthcare costs worldwide [1]. Despite improved acute survival due to rapid reperfusion, surviving patients are at risk of progression to heart failure [2]. Conventional long-standing drug therapy often ameliorates the chronic remodeling of the left ventricle to spare contractile function through interference with the neurohumoral system. Yet, the early inflammatory response to MI is now well recognized as a therapeutic target [3]. This physiologic response in the first hours to days after MI is a critical time period to determine adequate healing [3].

Specifically, inflammation after MI involves the dynamic migration of leukocytes, particularly macrophages, to the damaged area [4]. Early infiltrating cells including granulocytes and pro-inflammatory monocytes and macrophages mediate removal of cellular debris [5, 6]. Later, the predominant cell populations express reparative markers, which contribute to matrix remodeling and the formation of a stable collagen-rich scar [5]. In addition, during systemic inflammation post-MI, the leukocyte content is dominated by infiltrating leukocytes mobilized from hematopoietic organs, with a supplementary involvement of resident macrophages [7]. The severity of acute inflammation is inversely proportional to late functional outcome, but optimal healing requires a balance of inflammatory leukocytes to allow clearance of damaged cells and mediate repair and stable scar formation [8]. The goal of therapeutic strategies in the early post-MI phase is therefore to modulate inflammation in order to achieve optimal healing.

But cardiovascular disease is also associated with higher risk of cognitive impairment, dementia, and Alzheimer’s disease [9, 10]. Shared risk factors may contribute to this epidemiologic overlap, but we recently identified a contribution of heart–brain inflammatory crosstalk by demonstrating a direct neuroinflammatory response early after cardiac ischemia and in chronic heart failure [11, 12]. This biphasic neuroinflammation may contribute to subsequent neurodegeneration and cognitive decline as seen in Alzheimer’s disease [13, 14]. However, the precise mechanisms underlying this connection are not fully understood. Moreover, the reciprocal effects of anti-inflammatory therapies after MI on neuroinflammation are poorly characterized.

It has been shown that moderate doses of angiotensin-converting enzyme (ACE) inhibitors exhibit anti-inflammatory action besides their effects on neurohumoral activation [8] by interfering with excessive leukocyte mobilization from the spleen to the heart, and thereby contributing to improved contractile function after MI. In our prior work, continuous ACE inhibitor therapy lowered the acute and chronic cardiac and brain TSPO PET signals and spared cardiac function [12], but it remains unclear whether attenuated neuroinflammation derives from acute anti-inflammatory response to the therapy or chronic anti-remodeling action.

Accordingly, we aimed to evaluate the contribution of early enalapril therapy and delayed enalapril therapy on target cardiac inflammation and function, and on distant neuroinflammation. Serial non-invasive whole-body PET imaging of TSPO was employed to evaluate cardiac and neuroinflammation after coronary artery occlusion in mice under various treatment regimens, and was validated by autoradiography and histological tissue workup.

## Methods

### Study design

C57Bl/6 mice (*n* = 41) underwent coronary artery ligation (LAD-ligation) or sham surgery (*n* = 9). Permanent ligation was selected to maximize the inflammatory response after MI and ensure rapid progression to heart failure. MI mice were randomized to receive oral angiotensin-converting enzyme inhibitor enalapril (20 mg/kg/day) as an early preventative therapy (anti-inflammatory regimen; 10 days, beginning 3 days before MI surgery, *n* = 9) or as a delayed secondary therapy (anti-remodeling regimen; continuously from 7 days after MI, *n* = 16), or no therapy (*n* = 16). Sham animals received no treatment. This dose was selected based on previous experiments demonstrating mitigated leukocyte mobilization from the spleen [8]. Continuous administration of enalapril at this dose lowered cardiac and neuroinflammation after myocardial infarction [12]. The timing was chosen so that early treatment is restricted to the duration of the acute inflammatory phase, and delayed treatment starts after the expected resolution of post-MI inflammation. Longitudinal cardiac and neuroinflammation were interrogated non-invasively by TSPO PET imaging at 3 days, 7 days, and 8 weeks after MI.

Additional animals (*n* = 10) who did not receive PET or SPECT were sacrificed at 3 days and 7 days for ex vivo validation.

### Myocardial infarction

MI was induced by ligation of the left coronary artery as described [12]. Briefly, before surgery, mice were treated with analgesic butorphanol (2 mg/kg subcutaneously) and anesthetized with isoflurane (3% induction, retained 1.5 to 2% after oral intubation under mechanical ventilation). After left thoracotomy and opening of the pericardium, a ligature was secured around the left coronary artery. Sham surgery was performed identically, without securing the ligature.

### Radiochemistry

^18^F-GE180 was synthesized by semi-automated route in a radiochemistry module, as described previously [12], with high radiochemical purity, yield, and specific activity (450–600 GBq/μmol).

### Whole-body small-animal PET

Mice underwent serial PET imaging with ^18^F-GE180 using an Inveon DPET (Siemens, Knoxville, TN) as described previously. Briefly, mice were anesthetized with isoflurane (1.5–2.0%, 0.6 L/min O_2_). ^18^F-GE180 (14 ± 2 MBq) was administered via a catheter inserted into a lateral tail vein, and a dynamic 60-min image was acquired in listmode [12]. For anatomical coregistration of the PET signal, low-dose computed tomography (CT) was conducted afterwards. Images were histogrammed to 32 frames of 5 × 2, 4 × 5, 3 × 10, 8 × 30, 5 × 60, 4 × 300, and 3 × 600 s. Images were reconstructed to a 256 × 256 × 159 matrix (0.39 × 0.39 × 0.80 mm) with a 3D ordered subset expectation maximization/maximum a posteriori algorithm (*β* = 0.01, OSEM iterations = 2, MAP iterations = 18). Scatter and decay correction were implemented following manufacturer defaults.

### Cardiac function

To determine infarct size and assess contractile function, electrocardiographically gated perfusion SPECT scans were acquired as described previously [15]. Briefly, under isoflurane anesthesia, mice were injected with ^99m^Tc-sesatamibi (116 ± 13 MBq) as a bolus via a catheter inserted into a lateral tail vein. After 30 min of distribution, mice were positioned prone on the animal bed and ECG-gated listmode images were acquired using the Explore speCZT (TriFoil Imaging) equipped with a full ring of cadmium–zinc–telluride (CZT) detectors and a mouse 7-pinhole collimator. Images were acquired over ~ 25 min as described [12, 15], and reconstructed as a summed image and into 8 cardiac gates for functional analysis using an iterative algorithm. Additional details are provided in the online supplement.

### Image analysis

Cardiac PET images were analyzed with the Inveon Research Workplace software (Siemens). Brain PET images were analyzed using PMOD 3.7 software (PMOD Technologies Ltd., Zurich, Switzerland). For heart analysis, regions of interest (ROIs) were semi-automatically defined for the whole heart, the infarct region, and the remote myocardium. Cardiac data were analyzed semi-quantitatively as the average percent injected dose per gram of tissue (%ID/g) at 50–60 min after tracer injection. Cardiac ^99m^Tc-sestamibi distribution was evaluated by generating polar maps of coregistered SPECT and CT images using the Munich Heart software. Cardiac perfusion was normalized to the maximum activity excluding liver spillover. The perfusion defect was defined using a threshold (< 60%) of the normalized maximum signal. For differences in tracer delivery, the ^18^F-GE180 signal in the heart was normalized to relative perfusion in the hypoperfused territory compared with the normally perfused remote myocardium. Brain TSPO signal was determined by coregistering PET images to an MRI template and application of a brain atlas (mouse mirrone T2) to define a ROI for the whole brain. The final two frames of the dynamic image (40–60 min) were summed and uptake (%ID/g) was calculated. ROIs were defined by interactive thresholding in the spleen and bone marrow.

### Ex vivo validation

To corroborate in vivo PET images, mice were sacrificed for autoradiography and histologic analysis of cardiac and brain tissue at 3 days, 7 days, and 8 weeks post-MI. Mice were killed by cervical dislocation. Hearts were harvested and perfused with ice-cold PBS and snap-frozen in TissueTek. Brains were frozen in isopentane. Cardiac cryosections were sliced with 4 μm in the short axis at 200-μm increments. Brain cryosections were sliced with 14 μm at + 0.14, − 1.94, and − 3.16 according to Bregma. Sections were transferred on Superfrost+ microscope slides for further experiments [12]. Morphology and infarct scar were assessed by Masson trichrome staining. Immunostaining for Iba1-positive microglia, CD68-positive macrophages, Ly6G-positive granulocytes, and TSPO was conducted as described [12]. Additional details are provided in the online supplement.

### Statistics

All data are presented as mean ± standard deviation. Statistical analysis was performed using GraphPad Prism 7.0 (GraphPad Software). Measurements of in vivo and ex vivo data were compared by one-way analysis of variance with Tukey’s post hoc test for multiple comparisons. Pearson product-moment correlation coefficient evaluated linear regression between variables. Statistical significance was considered at *p* < 0.05.

## Results

### Early enalapril treatment attenuates myocardial TSPO PET signal post-MI

**Fig. 1:**
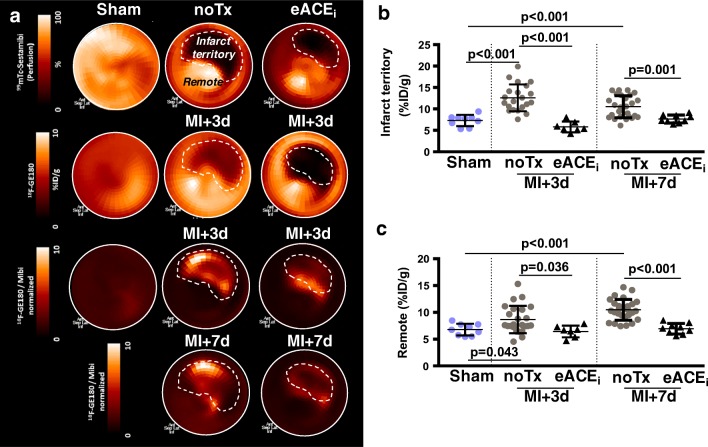
Acute anti-inflammatory effect of early enalapril therapy on heart after MI. **a** Representative polar maps of left ventricular ^99m^Tc-sestamibi perfusion (upper) and TSPO signal (middle) in sham and MI with and without early enalapril treatments. TSPO signal normalized to perfusion (both lower panels) shows elevated signal in the infarct territory of MI compared with sham, attenuated by enalapril. Semi-quantitative analysis of perfusion-normalized TSPO signal in **b** infarct territory and **c** remote myocardium. noTx, untreated; eACE_i_, early enalapril

Coronary artery ligation resulted in a perfusion defect covering 28% ± 15% of the left ventricle as determined by ^99m^Tc-sestamibi SPECT (Fig. 1a). Despite significant hypoperfusion, relative ^18^F-GE180 uptake was elevated in the infarct territory of untreated MI compared with that in sham animals (Fig. 1b), with a modest elevation in the remote myocardium (Fig. 1c). Elevated myocardial signal was associated with higher TSPO PET signal in hematopoietic reservoirs including the spleen (*r* = 0.482, *p* = 0.003) and bone marrow (*r* = 0.370, *p* = 0.027) (Online Fig. 1). Early treatment with enalapril significantly attenuated the perfusion-normalized TSPO PET signal in the infarct territory at 3 days and 7 days after MI (Fig. 1b). The remote non-infarct myocardial signal was also significantly lower with enalapril (Fig. 1c).

**Fig. 2:**
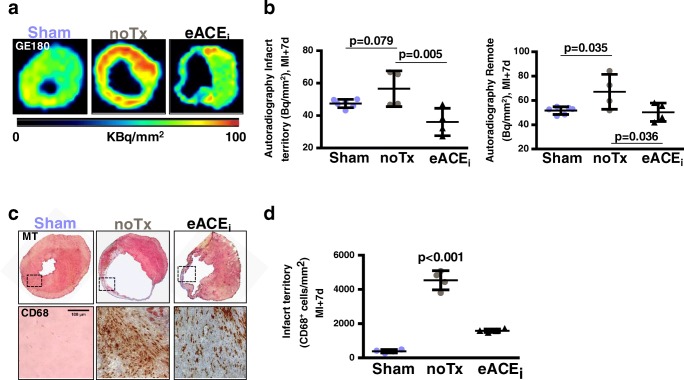
Correlative measurements of cardiac TSPO signal. **a** In vitro autoradiography heart sections and **b** quantitative analysis display elevated infarct activity compared with sham, attenuated by enalapril. **c** Immunostaining for CD68 shows accumulation of macrophages in the infarct wall defined by Masson trichrome (MT) staining. **d** Quantitative cell counting in histology demonstrates decreased macrophage content with enalapril therapy. noTx, untreated; eACE_i_, early enalapril

In vivo PET signals were validated by in vitro autoradiography (Fig. 2a), demonstrating a comparable twofold elevation in ^18^F-GE180 uptake in the infarct 7 days after MI to sham. The activity concentration was significantly reduced by enalapril therapy (Fig. 2b). Histology in adjacent sections displayed higher CD68^+^ macrophage content after MI that was attenuated by enalapril (Fig. 2c, d), but not in the remote myocardium (Online Fig. 2). Fluorescence co-immunostaining demonstrated colocalization of CD68^+^ cells with TSPO (Online Fig. 2).

### Early enalapril treatment attenuates acute brain inflammation after MI

**Fig. 3:**
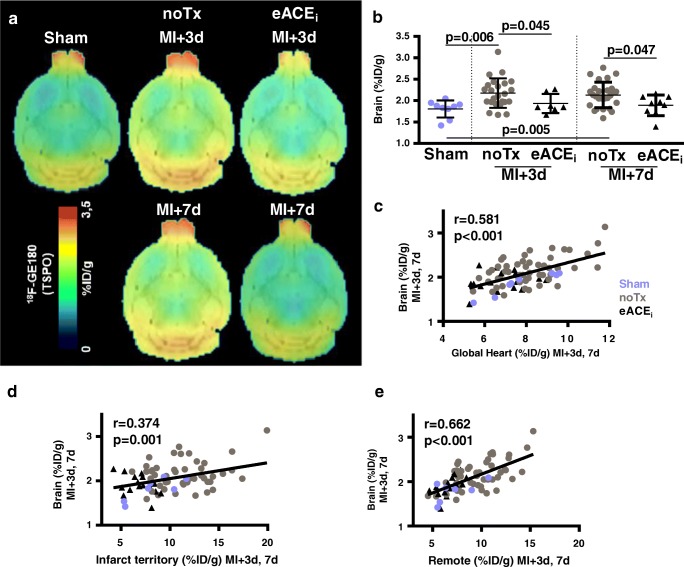
Acute anti-inflammatory effect of early enalapril therapy on neuroinflammation after MI. **a** Averaged brain images display increased TSPO signal after MI compared with sham, attenuated by enalapril. **b** Semi-quantification of brain TSPO signal post-MI. Brain TSPO signal early after MI directly correlates with TSPO signal in **c** global myocardium, **d** infarct territory, and **e** remote non-infarct myocardium. noTx, untreated; eACEi, early enalapril

Averaged brain images displayed higher global ^18^F-GE180 uptake in untreated animals in the first week after MI compared with sham (Fig. 3a, b). Early enalapril therapy significantly decreased global neuroinflammation defined by ^18^F-GE180 PET signal (Fig. 3b). Whole-brain TSPO PET signal within 1 week of MI correlated with global myocardial signal (*r* = 0.581, *p* < 0.001), the infarct territory signal (*r* = 0.374, *p* = 0.001), and remote non-infarct myocardial signal (*r* = 0.662, *p* < 0.001) (Fig. 3c–e). Moreover, the brain ^18^F-GE180 uptake correlated with hematopoietic spleen (*r* = 0.532, *p* < 0.001) and bone marrow TSPO signal (*r* = 0.345, *p* = 0.031) (Online Fig. 3).

**Fig. 4:**
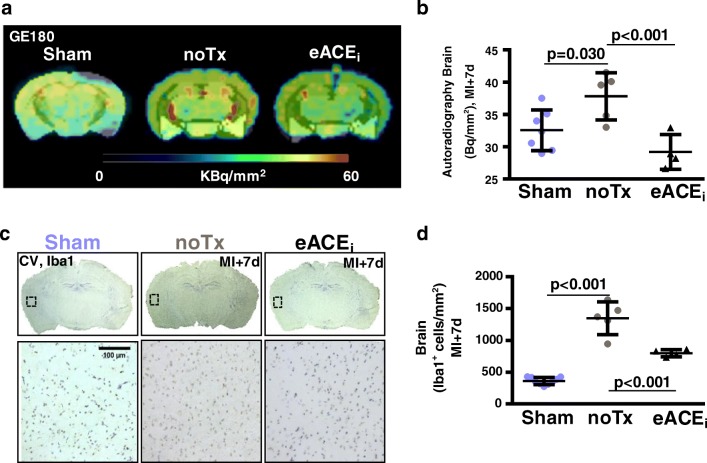
Correlative measurements of brain TSPO signal. **a** In vitro autoradiography brain sections and **b** quantitative analysis show elevated activity concentration at 7 days post-MI, attenuated by enalapril. **c** Corresponding Iba1 immunostaining identifies microglia in the cerebral cortex. **d** Quantification illustrates elevated Iba1 staining after MI which responds to early enalapril therapy. Counterstaining by cresyl violet (CV). noTx, untreated; eACE_i_, early enalapril treatment

In vitro autoradiography corroborated in vivo PET, displaying globally higher activity in the brain after MI compared with sham, which declined to sham levels with enalapril treatment (Fig. 4a, b). Quantitative histology indicated significantly higher Iba1+ microglial density after MI compared with sham, which was restored to sham levels by preventative enalapril (Fig. 4c, d). Fluorescence immunostaining demonstrated colocalization of Iba1^+^ microglia with TSPO, but not with GFAP^+^ astrocytes (Online Fig. 4).

### Early and delayed enalapril treatments lower global myocardial TSPO signal in chronic heart failure

**Fig. 5:**
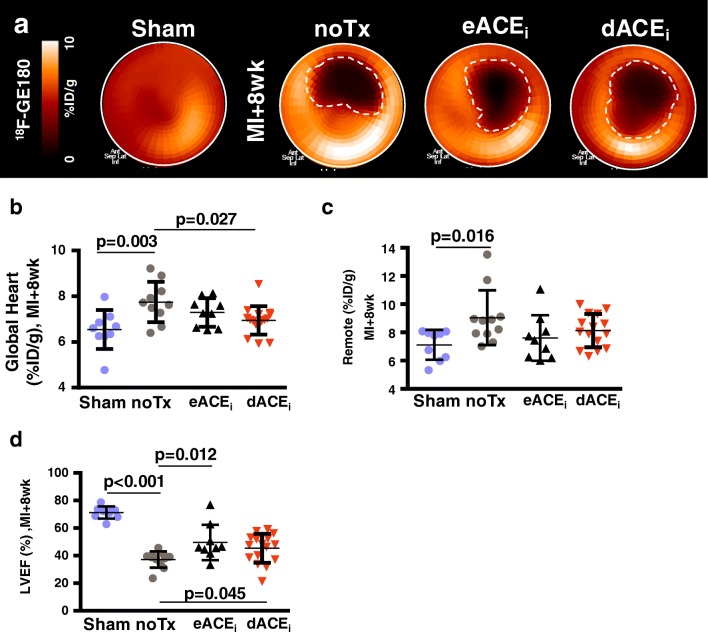
Chronic response of cardiac TSPO to enalapril therapy after MI. **a** Representative polar maps of sham and MI animals display elevated TSPO signal in failing non-infarct myocardium, partially attenuated by enalapril therapy. Quantitative analysis in **b** global and **c** remote non-infarct myocardium confirms the increase and treatment response. **d** Left ventricle ejection fraction (LVEF) at 8 weeks after MI is moderately improved by treatment with either early or delayed enalapril. noTx, untreated; eACE_i_, early enalapril treatment; dACE_i_, delayed enalapril treatment

Left ventricular polar maps demonstrate matched defect in ^18^F-GE180 uptake in the infarct territory at 8 weeks post-MI (Fig. 5a). Semi-quantitative analysis demonstrated the global myocardial TSPO signal was higher in untreated MI compared with that in sham (Fig. 5b), which was moderately and significantly reduced with delayed enalapril treatment, and tended to be lower with early enalapril treatment *(*Fig. 5b). The difference in TSPO signal derived predominantly from the remote myocardial territory (Fig. 5c).

**Fig. 6:**
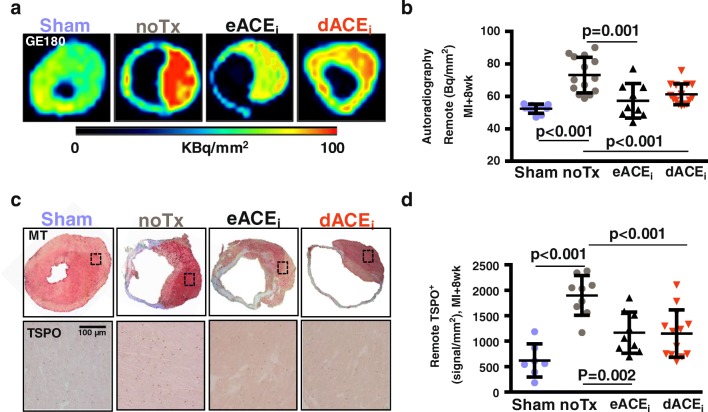
Correlative measurements of chronic cardiac TSPO signal. **a** Autoradiography heart sections and **b** quantitative analysis demonstrate higher TSPO signal in the remote myocardium after MI compared with those in sham, attenuated by early and delayed enalapril. **c** Immunostaining establishes TSPO localization to failing non-infarcted cardiomyocytes, which is partially alleviated by enalapril therapy. **d** TSPO signal quantification verifies visual analysis. noTx, untreated; eACE_i_, early enalapril treatment; dACE_i_, delayed enalapril treatment

This modest reduction in remote TSPO PET signal was more pronounced in quantitative in vitro autoradiography sections (Fig. 6a, b). Histology confirmed markedly higher TSPO staining in remote territory of MI hearts at 8 weeks compared with that in age-matched sham, which was lowered by both enalapril treatments (Fig. 6c, d). The ^18^F-GE180 signal intensity was comparable between in vivo PET and in vitro autoradiography (*r* = 0.497, *p* < 0.001), and between autoradiography signal and TSPO immunostaining (*r* = 0.462, *p* = 0.004) (Online Fig. 5).

### Early and delayed enalapril therapies and lower myocardial TSPO PET signal are associated with improved chronic cardiac function

Left ventricular ejection fraction was markedly reduced at 7 days and 8 weeks after infarction compared with sham (Fig. 5d and Online Fig. 6). Early or delayed treatment with enalapril led to improved ventricular function compared with untreated MI, with a tendency for greater improvement by early therapy. End-systolic and end-diastolic volumes were markedly elevated at 8 weeks after coronary ligation, but were not significantly reduced by enalapril therapy*.* Infarct size and heart mass were comparable between treatment groups (Online Fig. 6).

### Early and delayed enalapril treatments do not attenuate chronic neuroinflammation

**Fig. 7:**
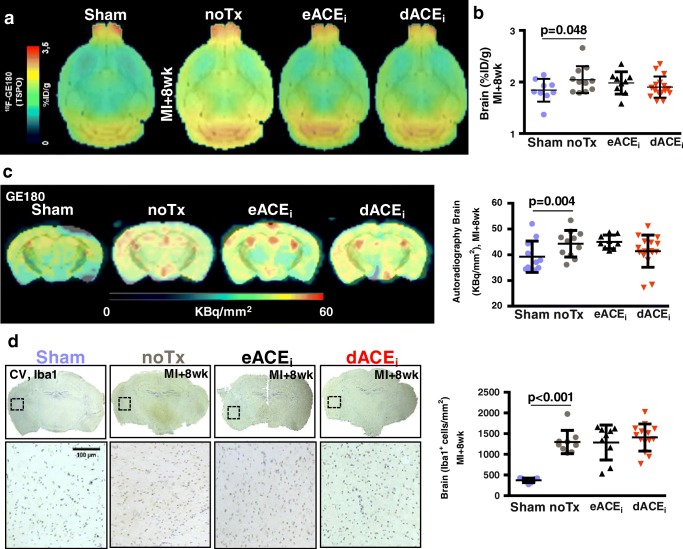
Chronic response of brain TSPO signal to enalapril therapy after MI. **a** Average coronal brain images and **b** semi-quantification display comparable TSPO signal in brain at 8 weeks after MI with and without enalapril. **c** In vitro autoradiography confirms globally elevated TSPO signal compared with sham. **d** Iba1 immunostaining identifies increased microglial content at 8 weeks after MI that remains elevated with enalapril therapy. noTx, untreated; eACE_i_, early enalapril treatment; dACE_i_, delayed enalapril treatment

Average brain images display elevated TSPO signal 8 weeks after MI (Fig. 7a). Uptake was modestly elevated in the global brain compared with that in sham, irrespective of enalapril treatment (Fig. 7b). The brain TSPO PET signal correlated with whole heart signal (*r* = 0.691, *p* < 0.001) and remote myocardial territory (*r* = 0.534, *p* < 0.001) (Online Fig. 7). Conversely, TSPO signal in the infarct region did not correlate with brain TSPO (Online Fig. 7). Chronic contractile function was modestly improved by enalapril when applied early or delayed, but did not result in a significant reduction of neuroinflammation PET signal. Brain TSPO signal at 8 weeks post-MI displayed a weak inverse correlation to ejection fraction (*r* = 0.298, *p* = 0.046), suggesting neuroinflammation may respond to contractile function.

In vitro autoradiography confirmed PET results, with a similar increase in global activity after MI refractory to enalapril (Fig. 7c). Histology identified comparable elevated Iba1^+^ microglia after MI regardless of treatment (Fig. 7d). As in the heart, the in vivo PET signal was proportional to in vitro autoradiography and Iba1+ microglia content (Online Fig. 8).

### Early cardiac inflammation predicts late cardiac function

**Fig. 8:**
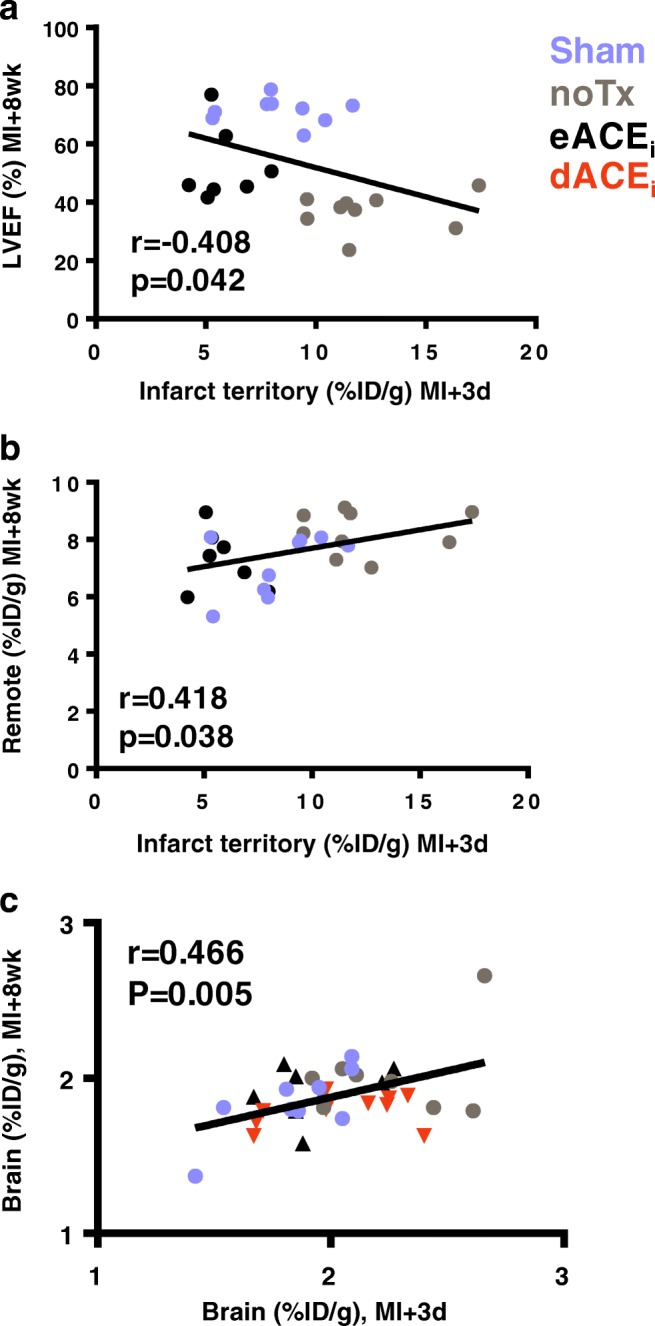
Prognostic value of PET inflammation signal in the heart and brain. **a** Infarct territory inflammation identified as perfusion-normalized TSPO signal 3 days after MI predicts subsequent contractile dysfunction in at 8 weeks. **b** Infarct territory inflammation predicts TSPO content in remote myocardium at 8 weeks. **c** Early ^18^F-GE180 uptake at 3 days post-MI predicts chronic brain ^18^F-GE180 uptake. noTx, untreated; eACE_i_, early enalapril treatment; dACE_i_, delayed enalapril treatment

Multiple regression analysis in all animals demonstrated that infarct territory TSPO signal at 3 days predicts cardiac function at 8 weeks (*r* = − 0.408, *p* = 0.042) (Fig. 8a). A weak correlation was observed for ventricular diameter but did not reach statistical significance (Online Fig. 9). TSPO signal in the infarct territory at 3 days post-MI correlated with late remote myocardial TSPO signal (*r* = 0.418, *p* = 0.038), supporting a relationship between acute inflammation and chronic cardiomyocyte mitochondrial dysfunction (Fig. 8b). Likewise, the early brain TSPO signal at 3 days post-MI was proportional to the chronic brain TSPO signal at 8 weeks (*r* = 0.466, *p* = 0.005) (Fig. 8c), suggesting that early microglial activation may predispose the brain to later dysfunction during heart failure.

## Discussion

Using whole-body TSPO-targeted molecular imaging, we previously demonstrated that myocardial infarction imparts concomitant cardiac and neuroinflammation early after the insult, with recurrent neuroinflammation in chronic heart failure. In this prior study, continuous treatment with angiotensin-converting enzyme inhibitor enalapril lowered the acute inflammation in the heart and brain, as well as chronic neuroinflammation in parallel with improved function [12]. Current clinical practice suggests that chronic therapy with ACE inhibitors attenuates adverse ventricular remodeling, but the impact of such treatment beyond the heart remains equivocal. In the present study, we demonstrate that the impact on neuroinflammation of ACE inhibitor therapy after myocardial infarction requires acute application and is directly related to the severity of inflammatory activity in the heart. Late application of therapy, omitting the acute anti-inflammatory effects, does not reduce chronic neuroinflammation, providing insights into the mechanistic interaction between acute cardiac inflammation, chronic heart function, and brain health. Serial whole-body ^18^F-GE180 PET in mice after MI identifies cardiac and neuroinflammation and TSPO as a marker for cardiac remodeling.

ACE inhibitors are a standard heart failure medication, targeting a range of physiologic processes to mediate adverse remodeling and improve cardiac function by reducing pre- and afterload, and dampening neurohumoral activation upstream of the myocardium to improve ejection fraction [16, 17]. More recently, ACE inhibitor therapy in mice was established to abolish the release of spleen-derived monocytes into the circulation, resulting in lower inflammatory cell infiltration to the infarct territory after coronary artery occlusion [8]. Reduced CD11b cell content was paralleled by a reduction of pro-inflammatory markers including Ly6C, TNF-α, and CD68 [8]. Indeed, angiotensin II has pro-inflammatory activity [18], and its suppression via ACE inhibitors or angiotensin receptor blockers lowers the production of IL-6 [19]. Continuous treatment with moderate-dose enalapril from 2 days prior to 8 weeks after coronary ligation evoked a decline in TSPO PET image–derived inflammation in the infarct territory, and confirmed improved chronic contractile function [8, 9]. However, the contributions of the acute anti-inflammatory benefit of enalapril on late ventricular function relative to chronic benefits on remodeling are not definitive. In the present study, both early and delayed ACE inhibitor therapies improved ejection fraction and attenuated ventricle dilatation at 8 weeks, suggesting an independent benefit of early anti-inflammatory therapy for late function. Indeed, acute ACE inhibitor application lowered inflammatory TSPO PET signal proportional to CD68 cell infiltration into the infarct territory. Notably, the improved cardiac function at 8 weeks was associated with lower chronic TSPO signal from the remote territory, which, due to the localization of the target to mitochondria, may provide an indirect indication of mitochondrial function and metabolism.

In chronic heart failure, the TSPO PET signal is elevated in the remote myocardium, confirming previous results [12], but the cellular basis of this signal is difficult to define. Here, immunostaining identified punctate intracellular TSPO expression in cardiomyocytes, reflected by semi-quantitative increase in the staining area in the remote myocardium. ACE inhibitor therapy is known to reduce oxidative stress [20], which may normalize oxidative metabolism and mitochondrial function in surviving cardiomyocytes. As such, TSPO PET imaging in chronic heart failure appears to provide a surrogate measurement of mitochondrial density which may be helpful for assessing ventricular remodeling and response to heart failure therapies. The precise relationship between TSPO expression and mitochondrial stress requires further evaluation, e.g., applying direct anti-oxidant therapy. Increased TSPO expression has been reported in the failing mouse heart after transverse aortic constriction, proportional to impaired mitochondrial energetics [21]. Angiotensin II has been suggested to regulate TSPO expression during stress, including in the heart [22]. The response to enalapril may be partially explained by this interaction, though we cannot exclude that the overall improvement in cardiac function also influences the TSPO signal independent of direct regulation.

The mechanisms underlying the response to ACE inhibitor therapy are largely related to cardiac function. With early treatment, modulated inflammation reduces adverse remodeling, leading to improved contractile function at 8 weeks. Conversely, delayed therapy impacts sympathetic neuronal activation and late remodeling, culminating in improved contractile function. The end result is lower stress on the myocardium, which may reduce mitochondrial dysfunction and explain the decline in TSPO signal. While limited to enalapril therapy in the present study, other cardiac interventions may have comparable ancillary benefits.

Inter-organ communication, the heart–brain axis, and systems biology are increasingly recognized as important contributors to the copresentation of disease in the aging population [11, 23]. We have previously established concomitant neuroinflammation after acute myocardial infarction and in chronic heart failure which was attenuated by continuous enalapril therapy [12]. Notably, the present study shows no reduction of TSPO PET signal or microglial content in the whole brain late after myocardial infarction with either early or delayed enalapril therapy. Nonetheless, chronic brain TSPO signal correlates with contractile function, with rising neuroinflammation corresponding to greater decline in ejection fraction. This observation suggests that despite persistent neuroinflammation with early or delayed enalapril therapy alone, neuroinflammation is inherently tied to the progression of heart failure. Worse contractile function translates to recurrent neuroinflammation, independent of inflammatory cell content in the myocardium. This microglial activation may occur in response to systemic cues such as impaired blood flow [24], compensatory neurohormonal activation [25], or systemically elevated inflammatory cytokines [26]. Further treatment studies should evaluate the contributions of such pathogenetic processes.

The chronic cardiac functional benefits of ACE inhibitor therapy appear to derive from both acute anti-inflammatory and chronic anti-remodeling benefits, as evidenced by improved ejection fraction with either early or delayed therapy. Neither early nor delayed ACE inhibitor therapy alone alleviated neuroinflammation, in contrast to our previous observation that continuous enalapril treatment lowered both the acute and chronic TSPO PET signals [12]. This suggests that therapeutic strategies may differ between the target and distant organs. Accordingly, higher dose or continuous enalapril administration may be necessary to optimize anti-remodeling efficacy. Alternatively, this discrepancy may reflect the limitations of gated perfusion SPECT for assessment of ventricle volumes in mice, which may be more accurately measured by dedicated anatomic modalities [15]. It should be noted that the enalapril dose used in the present study is potentially high relative to clinical doses. A dose–response study could provide additional translational insights.

The presence of neuroinflammation in chronic heart failure may derive from various contributing factors, including reduction of cerebral blood flow [27], heightened immune response [28], or whole-body oxidative stress [29]. The inverse relationship of chronic neuroinflammation to ejection fraction suggests that contractile dysfunction contributes to late microglial activation, but the precise mechanism warrants further investigation.

The early TSPO brain signal predicts late brain signal, suggesting that chronic neuroinflammation is influenced by acute microglial activation. Such an observation is consistent with the concept of central immune priming following a primary insult, as is observed in stroke or sepsis [30, 31]. In the pathogenesis of Alzheimer’s disease, microglial activation foreshadows subsequent neurodegeneration, prefacing a secondary wave of inflammatory activation in response to accumulating amyloid-β [32]. A recent study demonstrated that a single exposure to systemic lipopolysaccharide evoked acute neuroinflammation and exacerbated amyloid-β plaque development in a slow-developing Alzheimer’s disease mouse model [31]. Accordingly, the acute inflammatory activation following myocardial infarction may bear grave consequences for distant organs, including the brain, which may benefit from acute anti-inflammatory therapy. Previous studies identified elevated pro-inflammatory cytokines in the brain in mice with chronic heart failure which was associated with increased microglial activation and impaired cognitive performance [26]. Patients in chronic heart failure also exhibit higher levels of pro-inflammatory cytokines including TNF-α, IL-6, IL-1β, and C-reactive protein [33]. Inflammatory cytokines are known to cross the blood–brain barrier [34]. Accordingly, therapies targeted to upstream inflammatory mediators such as interleukin-1β, as shown in the Canakinumab CANTOS trial, may impart systems biology benefits following acute ischemic injury and in progressive heart failure [35, 36]. Interestingly, antibody-mediated suppression of IL-1β reduces the cerebral infarct size in a mouse stroke model [37], suggesting potential therapeutic opportunities with targeted agents. Consistent with this concept, the early anti-inflammatory enalapril therapy influenced neuroinflammation more definitively.

Some limitations of the present study should be considered. First, it should be noted that early and late ACE inhibitor therapies were not directly compared with continuous treatment, but with a historical dataset [12]. The duration between these experiments was minimal, and the same animal models and conditions were used to minimize fluctuation between groups. Second, the cognitive response to MI and enalapril therapy was not directly assessed. Nonetheless, previous evidence suggests impaired prefrontal memory in chronic heart failure mice [26], and the acute and chronic neuroinflammations are consistent with mouse models of cognitive impairment [38, 39]. Dedicated evaluations of cognitive outcomes after myocardial infarction are warranted. Third, the cellular basis of the TSPO PET signal is not completely defined. However, our immunostaining experiments demonstrate colocalization of TSPO with peripheral macrophages and microglia but not astrocytes in the acute phase, supporting specificity for inflammatory cells. This is consistent with experience in cell culture uptake assays [40]. Immunostaining of heart failure remote myocardium also suggests localization to mitochondria in cardiomyocytes, which aligns with the regulation of TSPO in heart failure [21]. Fourth, improvement of ejection fraction after enalapril therapy did not directly correspond to reduction in ventricular volumes. This may be related to the perfusion SPECT-derived calculation of ventricular geometry, which is subject to spillover from the ventricle wall to the lumen, or to the broader range of ventricle size typical of permanent left coronary ligation which limits statistical power [15]. Nonetheless, the modestly higher ejection fraction is consistent with prior reports using similar treatment [8, 12]. Finally, as TSPO PET primarily reflects inflammation, we have focused on these related mechanisms as influencing chronic ejection fraction and recurrent neuroinflammation. Other factors, such as brain perfusion or sympathetic neuronal activation [24, 25], may play a role in this observation, but require in-depth dedicated experiments.

In conclusion, whole-body TSPO PET imaging provides quantitative non-invasive indication of peripheral macrophages and central microglial activation after acute myocardial infarction, and altered mitochondrial function in chronic heart failure. Considering the previous evidence of reduced TSPO signal under continuous ACE inhibition, the functional benefits of ACE inhibitor therapy may capitalize on the combination of both acute anti-inflammatory effect and chronic attenuation of adverse remodeling, and may be required to alleviate chronic neuroinflammation, though further study is warranted.

## Electronic supplementary material

ESM 1 (DOCX 45 kb)

ESM 2 (PPTX 2555 kb)
