# Supplementary material for: Angiotensin-converting enzyme inhibitor treatment early after myocardial infarction attenuates acute cardiac and neuroinflammation without effect on chronic neuroinflammation
Source: Eur J Nucl Med Mol Imaging. 2020 Mar 3;47(7):1757–68. doi: 10.1007/s00259-020-04736-8 (PMC7248052; doi:10.1007/s00259-020-04736-8)
Supplement: Supplementary file 1 — (DOCX 45 kb) [file 259_2020_4736_MOESM1_ESM.docx]

**EXPANDED METHODS**

**Radiochemistry.** 10 µmol kryptofix 2.2.2, 10 µmol potassium bicarbonate and 0.5 mL acetonitrile (0.5 mL) were mixed with water and fluorine-18. The reaction mixture was heated to 100°C and dried under argon. Precursor mesylate (0.5-1 mg) was added in 1mL C2H3N and heated at 100°C for 10 min. The product was cooled and purified by semi preparative high performance liquid chromatography (ACE-5 C18 column). The purified ^18^F-GE180 was transferred onto a tC18 Sep-Pak cartridge and eluted with pure ethanol.

**Histology.** For histopathology, tissue sections were processed. Masson trichrome staining was performed to show cardiac morphology and scar formation and Cresyl violet staining was used to show brain morphology. Leukocyte infiltration was investigated by immuno histology staining CD68-positive macrophages using streptavidin horseradish peroxidase-diaminobenzidine. Neuroinflammation was investigated by immuno histology staining of Iba1-positive microglia cells using streptavidin horseradish peroxidase-diaminobenzidine. To determine colocalization of macrophages/microglia and TSPO, fluorescence immunostaining was performed for Brain sections using anti-TSPO antibodys and either anti-Iba1 for microglia or anti-GFAP for astrocytes, with DAPI staining. Remote myocardial TSPO density was determined by incubation of cardiac section with only anti-TSPO antibodys. Primary antibodies used were: rabbit anti-TSPO/peripheral benzodiazepine receptor (NBP1-45769, Novus Biologicals), AlexaFluor 488-conjugated rat anti-mouse CD68 (clone FA-11, MCA1957A488, Serotec), biotin-conjugated rat anti-mouse CD68 (clone FA-11, MCA1957B, BioRad). AlexaFluor 488-conjugated anti-GFAP (clone 2E1.E9, 644704, Biolegend) was used to stain astrocytes. Primary unconjuncated antibody (clone Poly-NB100, 1028SS, NovusBio) was used for Iba1. Delayed antibody used for TSPO immunostaining was AlexaFluor 594-conjugated donkey anti-rabbit IgG (clone poly4064, 406418, Biolegend) and delayed antibody used for Iba1 immunostaining was DyLight 488-conjugated donkey anti-goat IgG (clone poly ABIN2741791, Antibodies-Online). Fiji-Image J (http://imagej.nih.gov/ij/download.html) was used for quantitative histological analysis and image preparation.

**Autoradiography.** Autoradiography of the brain was performed as previously described [1]. Slices were incubated with ^18^F-GE180 for 30min, washed with PBS and water, and exposed to a high resolution imaging plate (PerkinElmer) for 30min in a light-impermeable cassette. A standard curve of known concentration was exposed in parallel for quantification. After exposure, images were digitized using a Cyclon scanner (PerkinElmer). Images were analyzed in PMOD 3.7 using a ROI for the whole brain and manually drawn ROI’s for hearts. Images were converted to a quantitative scale using the concurrently exposed standard curve of known concentration (Bq/mm^2^).

**ONLINE REFERENCES**

1. Thackeray JT, Derlin T, Haghikia A, Napp LC, Wang Y, Ross TL et al. Molecular Imaging of the Chemokine Receptor CXCR4 After Acute Myocardial Infarction. JACC Cardiovasc Imaging. 2015;8(12):1417-26. doi:10.1016/j.jcmg.2015.09.008.

**ONLINE FIGURE CAPTIONS**

**Online Figure 1. ^18^F-GE180 uptake by hematopoeitic organs after myocardial infarction.** (**a**) Spleen exhibits increased TSPO PET signal at 3d, 7d after MI compared to sham mice which is modestly lowered by early enalapril therapy. At 8wk no difference in splenic activity is observed compared to sham or untreated MI. (**b**) Bone marrow shows a similar moderate increase in TSPO PET signal early after MI, attenuated by acute enalapril therapy. No difference at 8wk is observed between groups. (**c**) Splenic and bone marrow (BM) TSPO signal correlate with infarct territory TSPO signal early after myocardial infarction.

**Online Figure 2. TSPO expression by CD68-positive macrophages in the infarct territory. (a)** CD68 immunostaining identifies increased macrophage infiltration in the infarct territory as shown by percentage of the field positively stained. (**b**) No difference in CD68 cell count or staining area is observed in the remote non-infarcted myocardium. (**c**) Co-immunofluorescence staining demonstrates co-localization of TSPO (red) with CD68 (green) in the infarct territory, showing selective expression of TSPO by infiltrating macrophages.

**Online Figure 3. Hematopoeitic organ activity early after MI correlates with neuroinflammation.** (**a**) Spleen and (**b**) bone marrow (BM) TSPO signal at 3d and 7d after MI directly correlate with global brain activity, suggesting a contribution of systemic inflammation to the acute neuroinflammatory response to MI.

**Online Figure 4. TSPO expression by Iba1-positive microglia in brain.** (**a**) Iba1 immunostaining identifies increased microglia in the cerebral cortex as shown by percentage of the field positively stained. (**b**) Co-immunofluorescence staining demonstrates co-localization of TSPO (red) with Iba1 (green, upper) positive microglia. No co-localization is observed between TSPO and GFAP (green, lower) positive astrocytes.

**Online Figure 5. Validation of in vivo PET to correlative measurements in heart.** (**a**) In vivo PET cardiac TSPO signal directly correlates with activity concentration defined by in vitro autoradiography. (**b**) In vitro autoradiography activity concentration in left ventricle correlates with CD68 positive macrophage content in the infarct territory.

**Online Figure 6. Contractile function at 8wk after coronary artery occlusion in mice.** (**a**) Left ventricle end systolic and (**b**) end diastolic volume derived from electrocardiogram-gated perfusion SPECT images display larger ventricle geometry at 8wk after MI compared to sham which is attenuated by early or delayed enalapril treatment. (**c**) Necropsy heart weight is elevated at 8wk after MI, partially reduced by enalapril therapy. (**d**) Perfusion defect extent as % of left ventricle displays comparable infarct size between treatment groups.

**Online Figure 7. Predictive factors of chronic brain TSPO signal.** Regression analysis between brain TSPO PET signal at 8wk and (**a**) global heart and (**b**) remote myocardium at 8wk show correlation with the heart and brain signals. (**c**) Brain activity does not correspond to the late infarct territory signal. (**d**) TSPO PET signal is inversely correlated to left ventricle ejection fraction (LVEF) at 8wk after MI.

**Online Figure 8. Validation of in vivo PET to correlative measurements in brain.** (**a**) In vivo PET brain TSPO signal directly correlates with activity concentration defined by in vitro autoradiography. (**b**) In vitro autoradiography activity concentration in cerebrum correlates with Iba1 positive microglia content in the cerebral cortex.

**Online Figure 9. TSPO PET may predict late ventricle remodeling.** Chronic remote non-infarcted myocardial TSPO PET signal show a trend to correlation with ventricular volume at (**a**) end systole and (**b**) end diastole.
