# Supplementary material for: Angiotensin-converting enzyme inhibitor treatment early after myocardial infarction attenuates acute cardiac and neuroinflammation without effect on chronic neuroinflammation
Source: Eur J Nucl Med Mol Imaging. 2020 Mar 3;47(7):1757–68. doi: 10.1007/s00259-020-04736-8 (PMC7248052; doi:10.1007/s00259-020-04736-8)
Supplement: Supplementary file 2 — (PPTX 2555 kb) [file 259_2020_4736_MOESM2_ESM.pptx]

## Slide 1
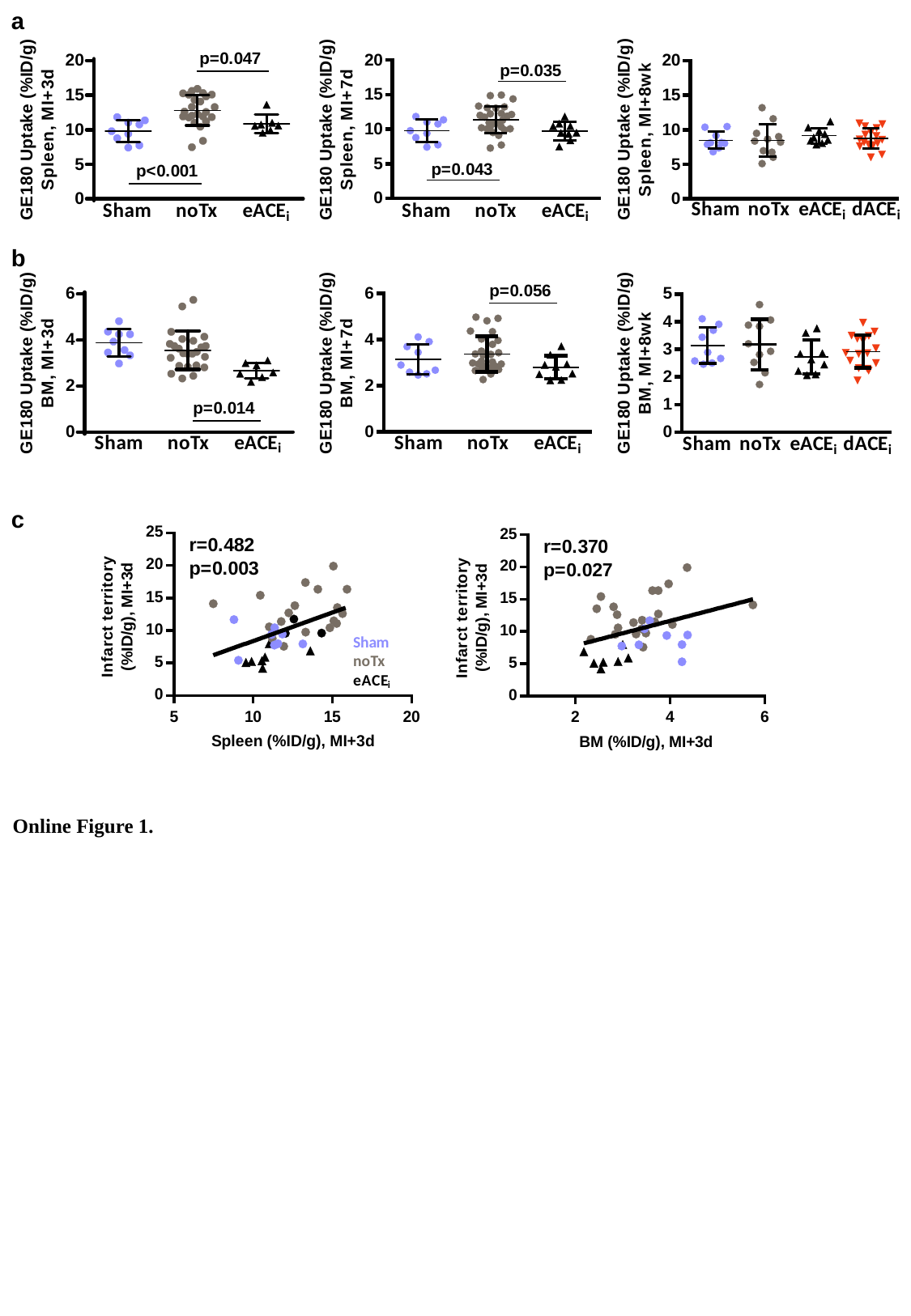

a
b
c
Online Figure 1.

## Slide 2
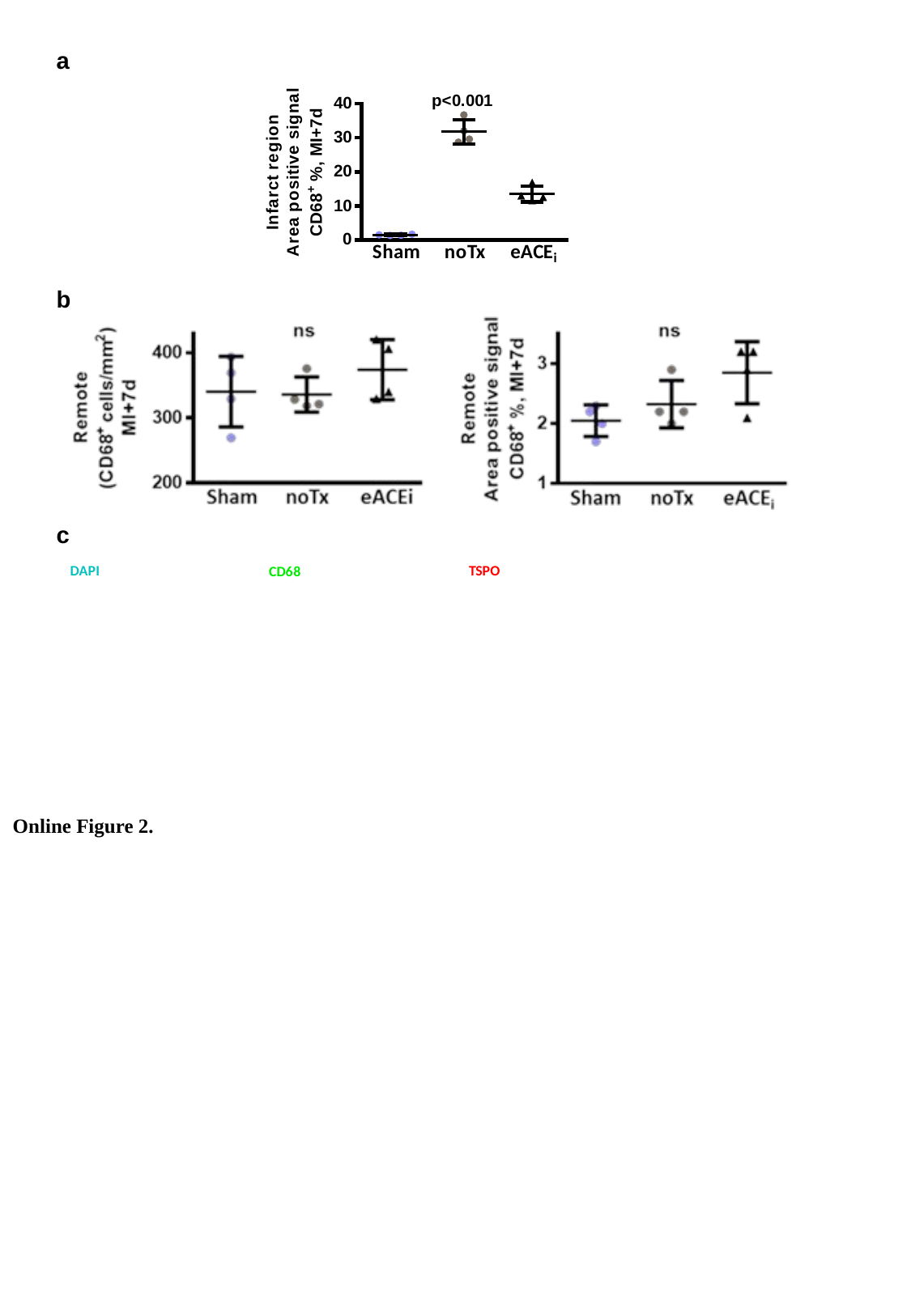

a
b
c
Online Figure 2.

## Slide 3
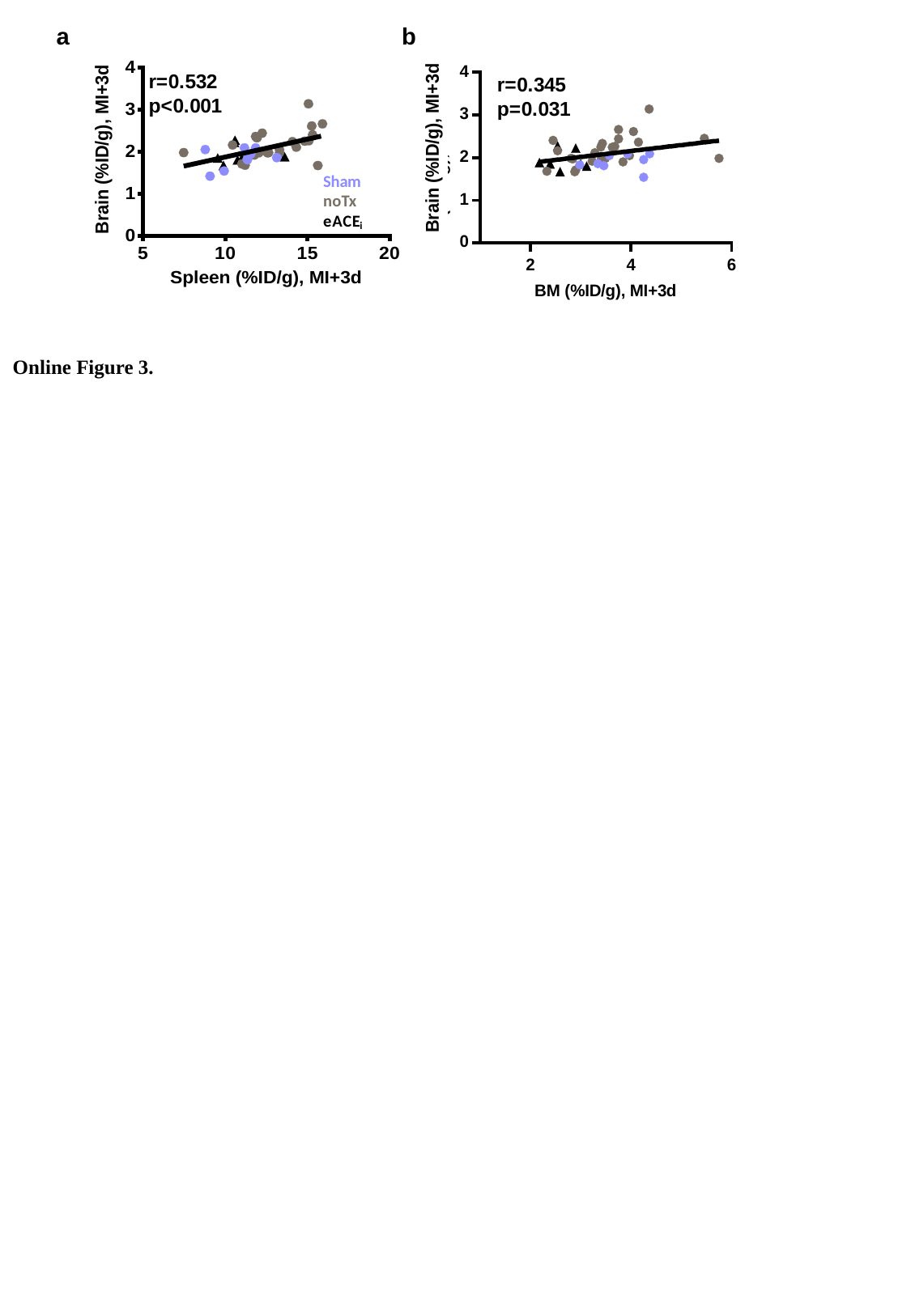

a
b
Online Figure 3.

## Slide 4
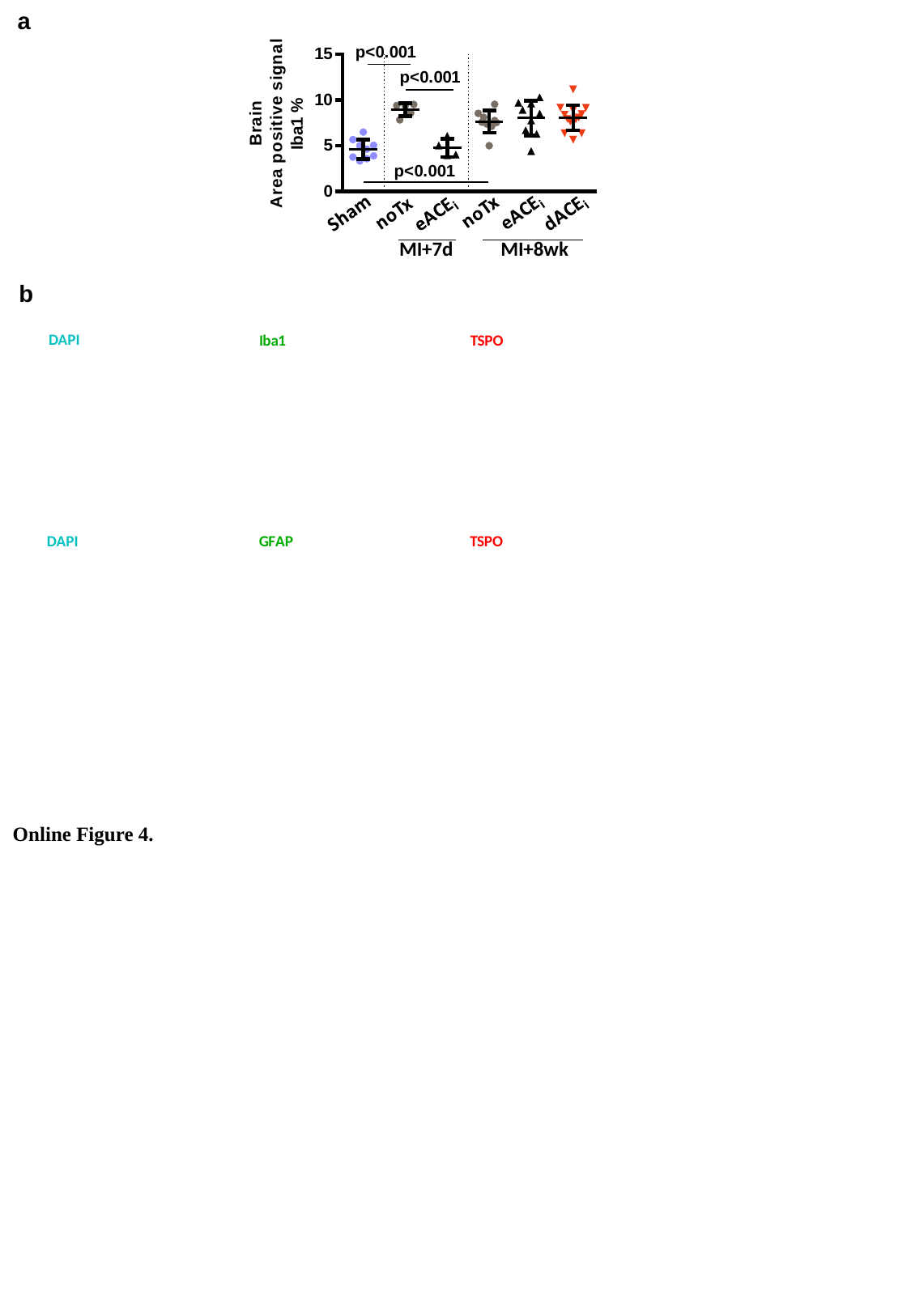

a
b
Online Figure 4.

## Slide 5
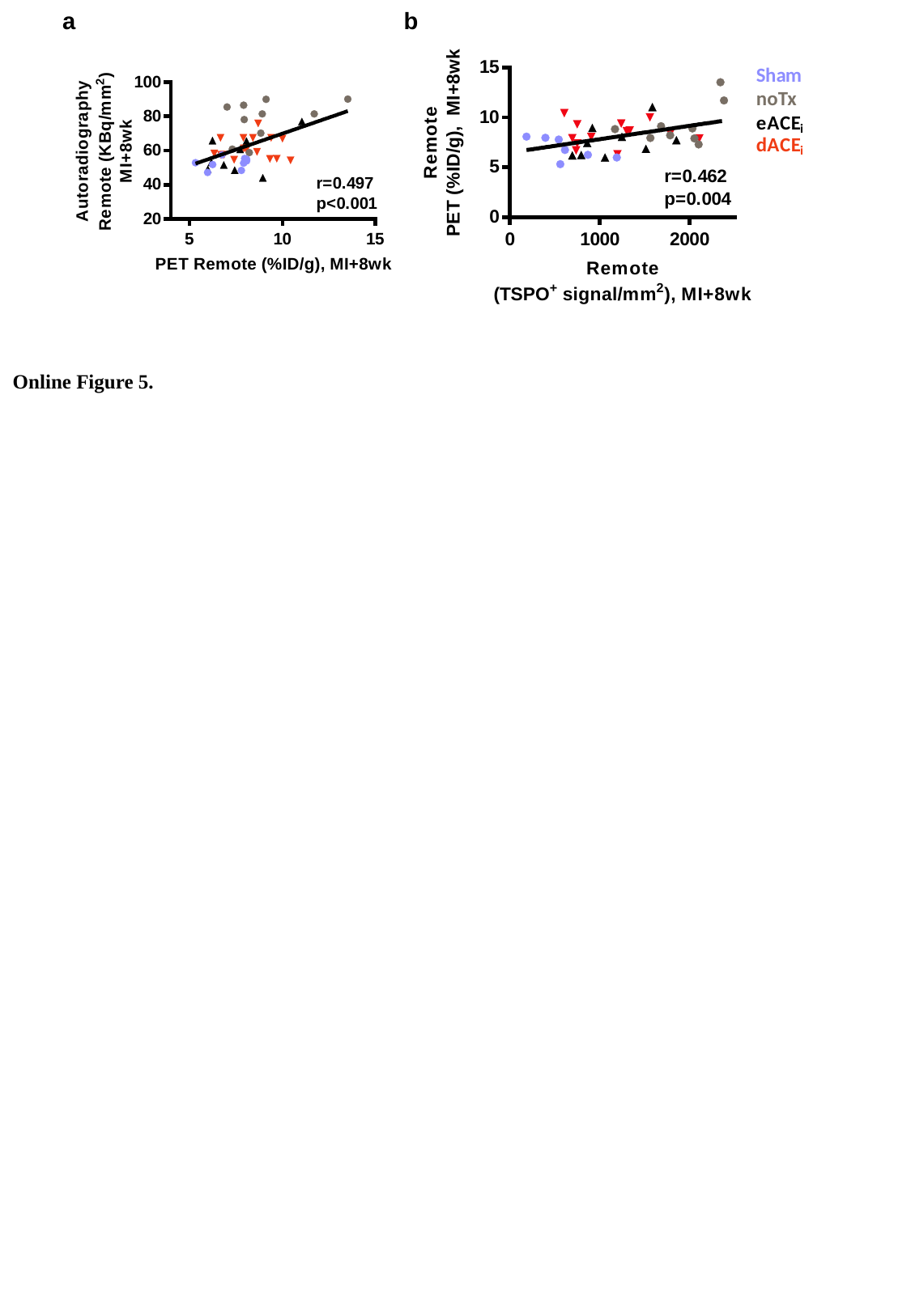

a
b
Online Figure 5.

## Slide 6
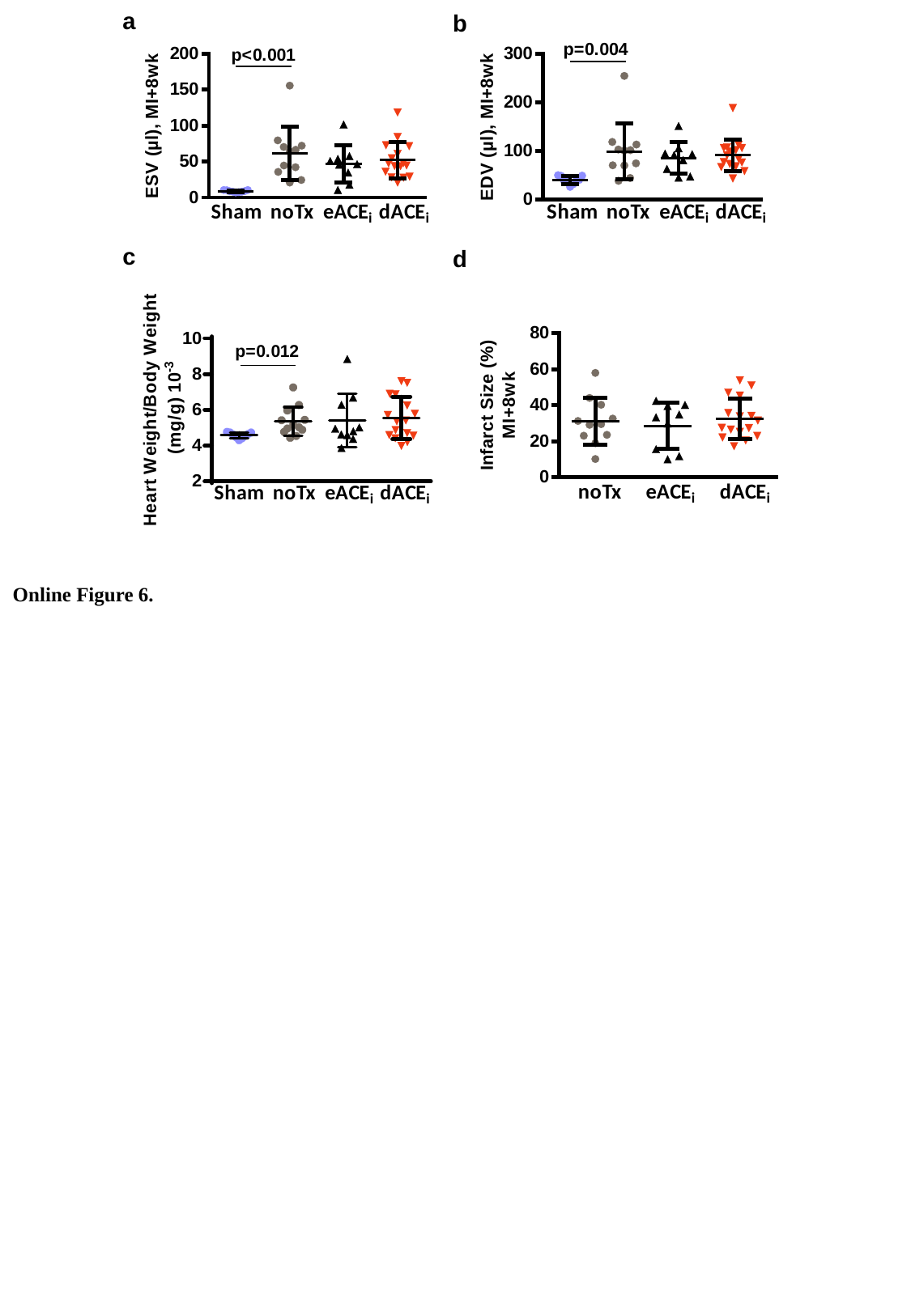

a
b
c
d
Online Figure 6.

## Slide 7
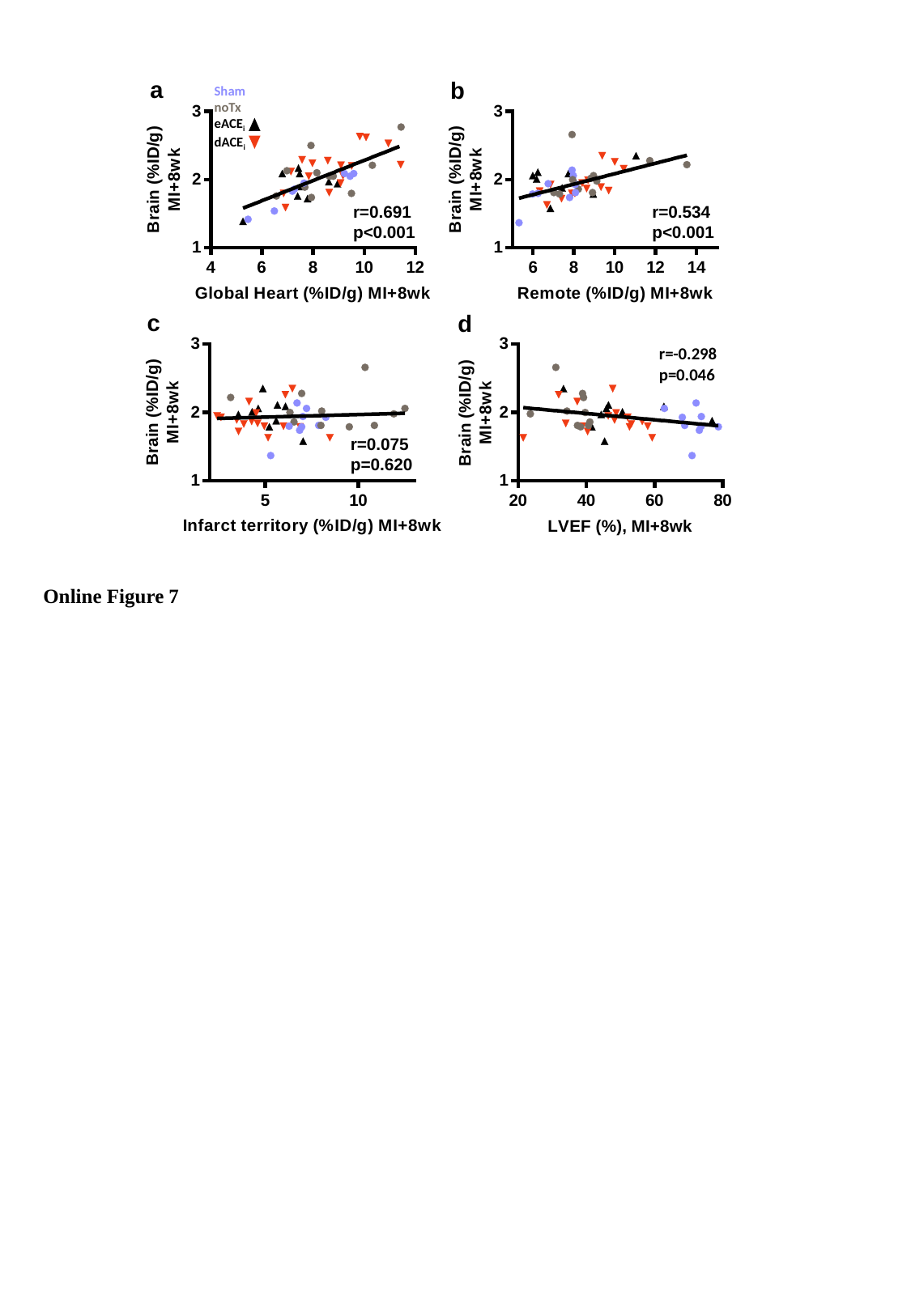

a
b
Sham
noTx
eACEi
dACEi
r=0.534
p<0.001
r=0.691
p<0.001
c
d
r=-0.298
p=0.046
r=0.075
p=0.620
Online Figure 7

## Slide 8
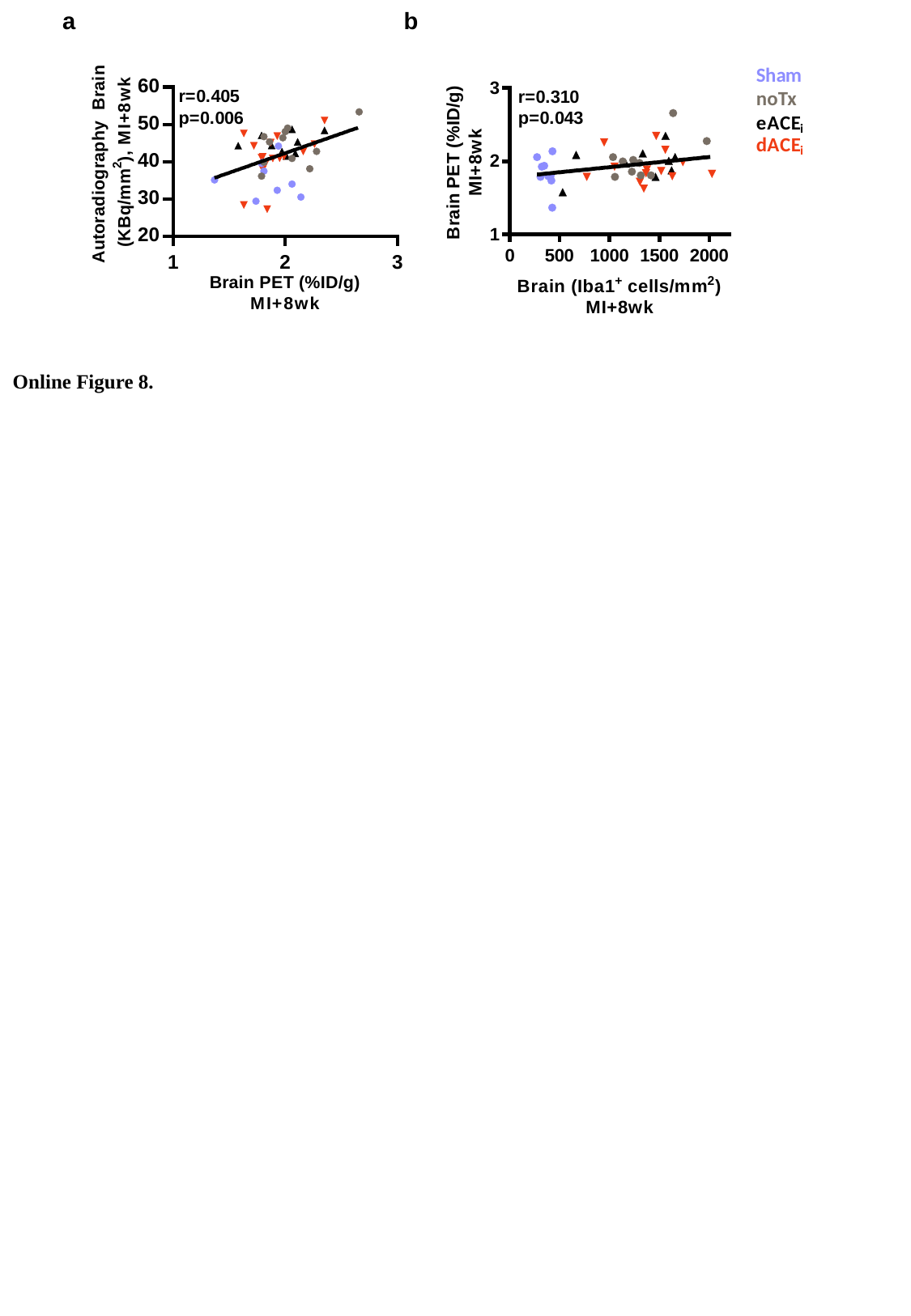

a
b
Online Figure 8.

## Slide 9
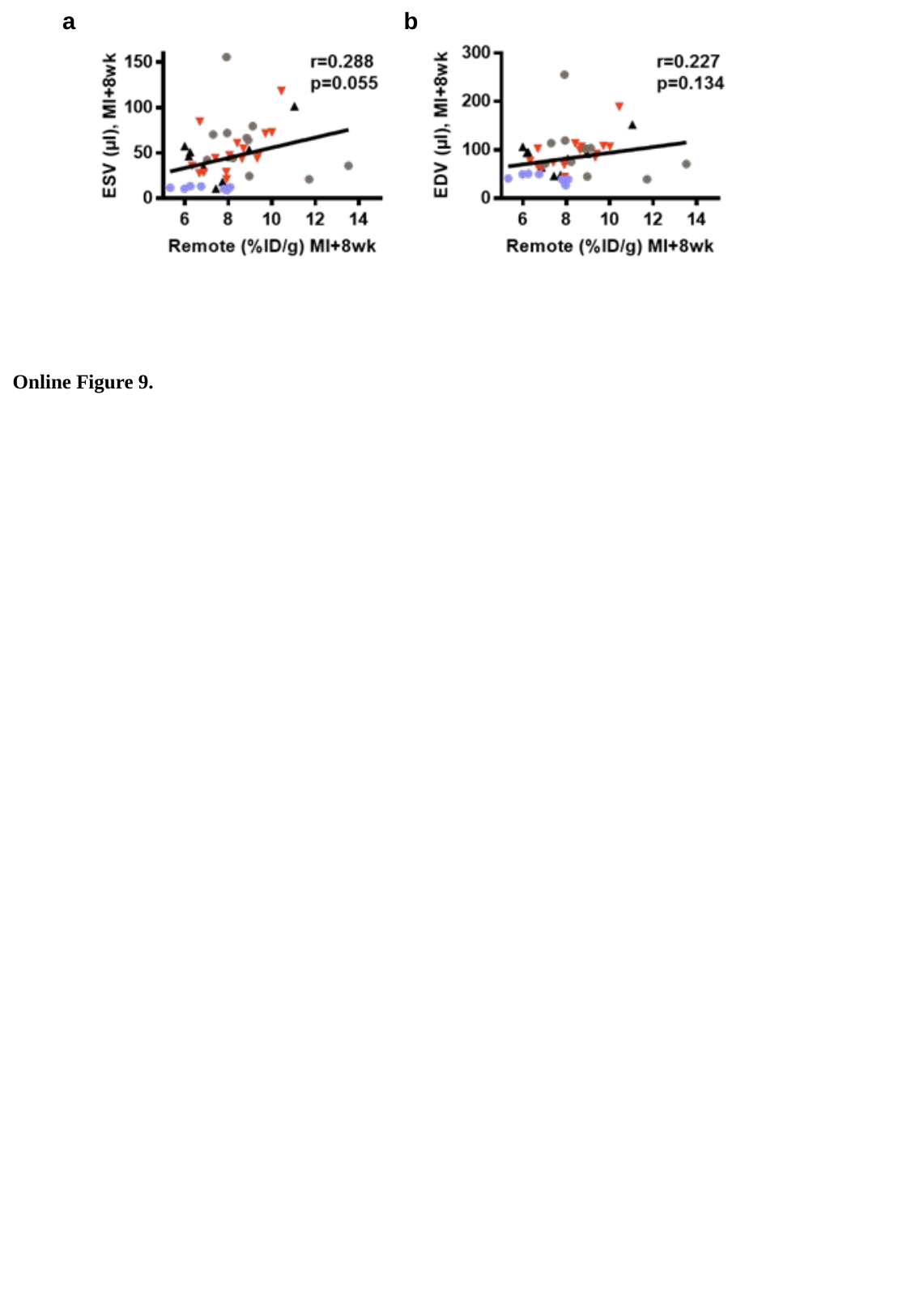

a
b
Online Figure 9.
